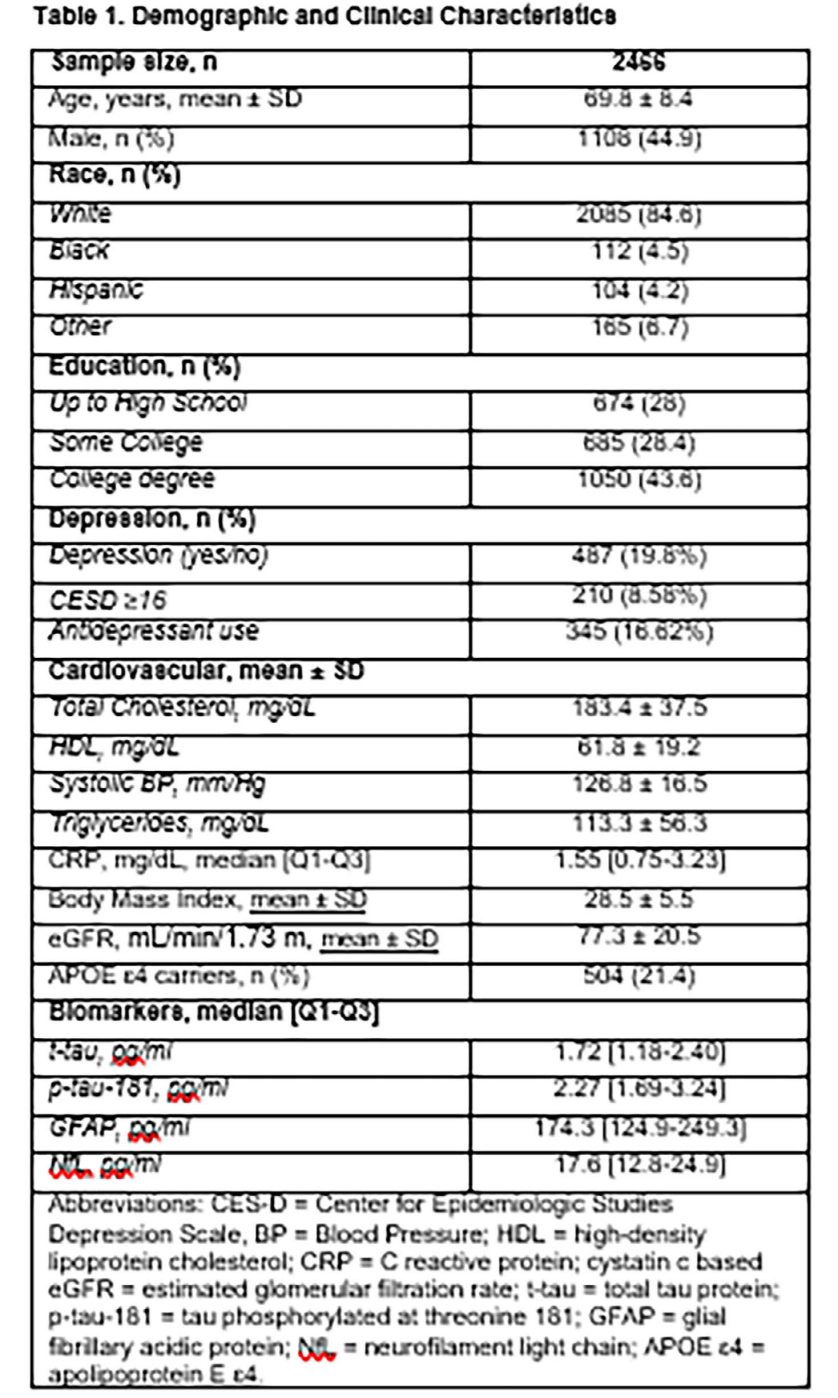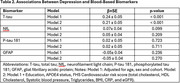# Depression is associated with higher t‐tau level in plasma: a report from the Framingham Heart Study

**DOI:** 10.1002/alz70856_103034

**Published:** 2025-12-26

**Authors:** Carlos A. Gaona, Vanessa M. Young, Crystal Wiedner, Arash Salardini, Agustin Ruiz, Rebecca Bernal, Alexa S Beiser, Jayandra Jung Himali, Sudha Seshadri, Antonio L Teixeira

**Affiliations:** ^1^ Glenn Biggs Institute for Alzheimer's and Neurodegenerative Diseases, University of Texas Health Science Center, San Antonio, TX, USA; ^2^ Graduate School of Biomedical Sciences, University of Texas Health Science Center, San Antonio, TX, USA; ^3^ School of Social and Behavioral Sciences, Arizona State University, Phoenix, AZ, USA; ^4^ Glenn Biggs Institute for Alzheimer's & Neurodegenerative Diseases, University of Texas Health Science Center, San Antonio, TX, USA; ^5^ Glenn Biggs Institute for Alzheimer's & Neurodegenerative Diseases, University of Texas Health Science Center, San Antonio, TX, USA; ^6^ Department of Neurology, University of Texas Health Sciences Center, San Antonio, TX, USA; ^7^ Joe R. and Teresa Lozano Long School of Medicine, University of Texas Health Science Center, San Antonio, TX, USA; ^8^ Biomedical Research Networking Centre in Neurodegenerative Diseases (CIBERNED), National Institute of Health Carlos III, Madrid, Madrid, Spain; ^9^ Ace Alzheimer Center Barcelona – International University of Catalunya (UIC), Barcelona, Spain; ^10^ Department of Microbiology, Immunology and Molecular Genetics, University of Texas Health Science Center, San Antonio, TX, USA; ^11^ Glenn Biggs Institute for Alzheimer's & Neurodegenerative Diseases, University of Texas Health San Antonio, San Antonio, TX, USA; ^12^ Boston University School of Public Health, Boston, MA, USA; ^13^ The Framingham Heart Study, Framingham, MA, USA; ^14^ Boston University Chobanian & Avedisian School of Medicine, Boston, MA, USA; ^15^ Department of Neurology, Boston University Chobanian & Avedisian School of Medicine, Boston, MA, USA; ^16^ Department of Population Health Sciences, UT Health San Antonio, San Antonio, TX, USA; ^17^ Glenn Biggs Institute for Alzheimer's &Neurodegenerative Diseases, University of Texas Health Science Center, San Antonio, TX, US., San Antonio, TX, USA; ^18^ Graduate School of Biomedical Sciences, University of Texas Health Science Center, San Antonio, TX, USA, San Antonio, TX, USA

## Abstract

**Background:**

Depression is an established risk factor for neurodegenerative diseases, including Alzheimer's disease (AD). While blood‐based biomarkers (BBMs) have been promising for early detection of neurodegeneration, their relationship with depressive symptoms remains controversial. We explored the cross‐sectional relationship between depression and BBMs of neurodegeneration and AD pathology (t‐tau, NfL, GFAP, and *p*‐tau181) using ultrasensitive immunoassay.

**Method:**

The current study comprises the Offspring (exam 9) and Omni 1 (exam 4) cohorts of the Framingham Heart study (FHS) who completed their visits between 2011 and 2014. Depression was considered a binary variable (yes/no) and defined as having a score ≥16 on the Center for Epidemiologic Studies Depression Scale or reporting antidepressant use. Single Molecule Array (SIMOA) assays were used to assess plasma levels of t‐tau, NfL, GFAP, and *p*‐tau181. All BBM values were log‐transformed and standardized. Multivariable regression models were adjusted for (1) sex, age, cohort, and additionally (2) education, APOE4 status, FHS cardiovascular risk score, estimated glomerular filtration rate, and C‐reactive protein.

**Result:**

Among 2,466 participants (mean age 69.8 years, 44.9 % male), 19.8% met criteria for depression (see Table 1 for clinical characteristics). Depression was significantly associated with higher t‐tau levels in both model 1 (β±SE =0.24±0.05, *p* <0.001) and model 2 (0.21±0.05, *p* <0.001). No associations were found between depression and *p*‐tau 181, NfL, or GFAP, as shown in Table 2.

**Conclusion:**

Depression was associated with higher t‐tau levels in these cohorts. While current findings corroborate previous studies showing that depressive symptoms were not associated with plasma biomarkers of AD pathology, the positive association with t‐tau level suggests the involvement of neuronal injury.